# In-depth genomic data analyses revealed complex transcriptional and epigenetic dysregulations of *BRAF*^V600E^ in melanoma

**DOI:** 10.1186/s12943-015-0328-y

**Published:** 2015-03-14

**Authors:** Xingyi Guo, Yaomin Xu, Zhongming Zhao

**Affiliations:** Department of Biomedical Informatics, Vanderbilt University School of Medicine, Nashville, TN 37203 USA; Division of Epidemiology, Department of Medicine, Vanderbilt Epidemiology Center, Vanderbilt-Ingram Cancer Center, Vanderbilt University School of Medicine, Nashville, TN 37232 USA; Department of Biostatistics, Vanderbilt University School of Medicine, Nashville, TN 37203 USA; Center for Quantitative Sciences, Vanderbilt University Medical Center, Nashville, TN 37232 USA; Department of Cancer Biology, Vanderbilt University School of Medicine, Nashville, TN 37232 USA

**Keywords:** Melanoma, Expression, DNA methylation, Driver mutation, *BRAF*, *MITF*, *TGFB1*, *DNMT3A*

## Abstract

**Background:**

The recurrent *BRAF* driver mutation V600E (*BRAF*^V600E^) is currently one of the most clinically relevant mutations in melanoma. However, the genome-wide transcriptional and epigenetic dysregulations induced by *BRAF*^V600E^ are still unclear. The investigation of this driver mutation’s functional consequences is critical to the understanding of tumorigenesis and the development of therapeutic strategies.

**Methods and results:**

We performed an integrative analysis of transcriptomic and epigenomic changes disturbed by *BRAF*^V600E^ by comparing the gene expression and methylation profiles of 34 primary cutaneous melanoma tumors harboring *BRAF*^V600E^ with those of 27 *BRAF*^WT^ samples available from The Cancer Genome Atlas (TCGA). A total of 711 significantly differentially expressed genes were identified as putative *BRAF*^V600E^ target genes. Functional enrichment analyses revealed the transcription factor MITF (p < 3.6 × 10^−16^) and growth factor TGFB1 (p < 3.1 × 10^−9^) were the most significantly enriched up-regulators, with *MITF* being significantly up-regulated, whereas *TGFB1* was significantly down-regulated in *BRAF*^V600E^, suggesting that they may mediate tumorigenesis driven by *BRAF*^V600E^. Further investigation using the MITF ChIP-Seq data confirmed that *BRAF*^V600E^ led to an overall increased level of gene expression for the MITF targets. Furthermore, DNA methylation analysis revealed a global DNA methylation loss in *BRAF*^V600E^ relative to *BRAF*^WT^. This might be due to BRAF dysregulation of *DNMT3A*, which was identified as a potential target with significant down-regulation in *BRAF*^V600E^. Finally, we demonstrated that *BRAF*^V600E^ targets may play essential functional roles in cell growth and proliferation, measured by their effects on melanoma tumor growth using a short hairpin RNA silencing experimental dataset.

**Conclusions:**

Our integrative analysis identified a set of *BRAF*^V600E^ target genes. Further analyses suggested a complex mechanism driven by mutation *BRAF*^V600E^ on melanoma tumorigenesis that disturbs specific cancer-related genes, pathways, and methylation modifications.

**Electronic supplementary material:**

The online version of this article (doi:10.1186/s12943-015-0328-y) contains supplementary material, which is available to authorized users.

## Introduction

Next-generation sequencing has enabled us to identify numerous genetic alternations in melanoma genomes. These genetic alterations provide us with opportunities not only to investigate the novel insights into the molecular mechanisms of melanoma tumorigenesis but also to provide a new discovery basis for the identification of biomarkers for personalized targeted therapies [1-3]. So far, several driver genes including *BRAF*, *NRAS*, *KIT*, *GNAQ*, and *GNA11* have been characterized and routinely used in clinical screenings for melanoma [4-6]. Other clinically relevant mutations or genes associated with those driver genes were systematically explored from 241 melanoma genomes [7]. Among these driver genes, the *BRAF* mutation at position 600 (*BRAF*^V600^) occurs in approximately 50% of melanoma patients, and among them, V600E accounts for approximately 79% [8]. The *BRAF*^V600^ in melanoma tumor genomes is currently one of the most clinically relevant mutation sites in melanoma [4,9]. Importantly, BRAF inhibitors such as vemurafenib and dabrafenib have been developed as targeted therapies for melanoma patients that harbor the *BRAF*^V600E^ mutation. These compounds have provided tremendous clinical benefit to personalized cancer treatment; unfortunately, like other inhibitors, patients eventually develop resistance after treatment [10-12].

The exploration of the functional consequences of the transcriptional dysregulations of *BRAF*^V600E^ is critical to the understanding of tumorigenesis and the potential discovery of targeted therapy. BRAF is part of the mitogen-activated protein kinase (MAPK) pathway that regulates cell growth and proliferation. The gain-of-function in *BRAF*^V600E^ is well-known to highly activate the MAPK kinase pathway that promotes tumor cell growths in melanoma [13]. In recent years, several groups have explored the downstream genes promoted by *BRAF*^V600E^ [14,15]. For example, Kannengiesser *et al.* [14] identified a few hundred genes associated with *BRAF*^V600E^ through the differential analysis of microarray gene expression data in a survey of 69 human primary cutaneous melanoma tumors. Interestingly, they observed that most of those *BRAF*^V600E^ regulated genes controlled by MITF were associated with over-expression. Through the investigation of transcriptome-wide changes using transduction *BRAF*^*V600E*^ on primary human melanocytes, Flockhart *et al.* [15] have recently reported approximately one thousand mRNA transcripts that may be impacted by *BRAF*^V600E^.

Accumulating evidence has shown that aberrant methylation leads to the initiation and progression of tumorigenesis and this has been recognized as a hallmark of cancer [16,17]. Aberrant methylation in specific cancer genes has been reported to contribute to melanoma development [18-20]. Although the gain-of-function of *BRAF*^V600E^ can promote specific target genes and pathways, to what extent the epigenetic modifications (i.e. DNA methylation) are involved and how to interplay within this process has been poorly understood.

The Cancer Genome Atlas (TCGA) project generated massive high-throughput genomic data, including mutation, DNA methylation, and transcription profiles for several hundred melanoma samples. These data provide us with an unprecedented opportunity for in-depth exploration of the functional consequences of a driver mutation (e.g., *BRAF*^V600E^) on tumors that integrate multiple types of genomic data. For this purpose, we performed an integrative analysis of the transcriptional and epigenetic alterations associated with a driver mutation (*BRAF*^*V600E*^) and applied it to the primary and metastatic tumor cutaneous melanoma samples available from TCGA.

## Results

### Differential gene co-expression analyses identified putative targets of *BRAF*^V600E^

**Figure 1:**
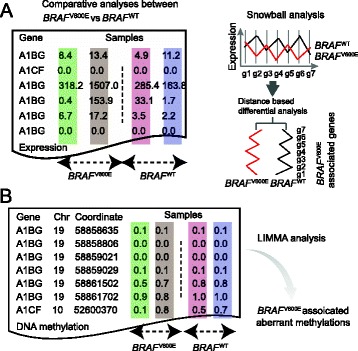
**Workflow for identifying significantly altered transcriptional and epigenetic regulations associated with the** ***BRAF*** ^**V600E**^ **driver mutation in melanoma.** Matched expression **A)** and methylation profiles **B)** are built for *BRAF* ^V600E^ and *BRAF* ^WT^ samples. Expression and methylation data in the column highlighted in the same color are derived from the same sample. We used the Snowball approach (top) to identify significantly and differentially expressed genes. A schematic demonstration of the Snowball approach for the identification of *BRAF* ^V600E^ putative targets is shown in the top right panel. Gene expression profiles for multiple cancer samples are measured in two groups, *BRAF* ^V600E^ and *BRAF* ^WT^. All genes (g1-g7) can be powerfully detected based on their co-expression profiles from the *BRAF* mutation and wild-type groups (for details see Materials and methods). The LIMMA method was applied to detect differential methylation loci between *BRAF* ^V600E^ and *BRAF* ^WT^ samples (bottom).

**Figure 2:**
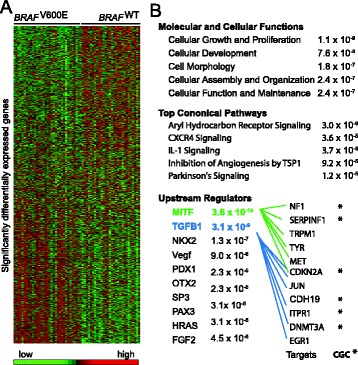
**Functional analysis of** ***BRAF*** ^**V600E**^ **target genes identified in primary tumor samples. A)** Heat-map showing the differential signals for *BRAF* ^*V600E*^ target genes identified by Snowball approach. **B)** Enriched functional categories of the *BRAF* ^*V600E*^ target genes. * refers to genes in CGC catalogue.

To identify the genes and related pathways perturbed by *BRAF*^V600E^, we developed a novel statistical approach, named Snowball, to identify differentially expressed genes based on their aggregated association between co-expression patterns and *BRAF*^V600E^ mutation status. We identified the regulatory network modules that were significantly associated with *BRAF*^V600E^ with a permutation p < 0.05, followed by a Weighted Gene Co-expression Network analysis (see Materials and methods, Figure 1A) [21]. As a result, a total of 711 putative target genes were identified including 330 down-regulated and 381 up-regulated genes (Additional file 1). Figure 2A shows a heat-map of expression patterns in the *BRAF*^V600E^ and *BRAF*^WT^ samples for those significantly associated genes identified by Snowball.

Next, we used Ingenuity Pathway Analysis (IPA) to examine the functional categories and biological pathways of those putative *BRAF*^V600E^ target genes. A significant portion of them were cancer-related (p < 1.6 × 10^−8^), including 20 genes from the Cancer Gene Census (CGC) catalogue (Figure 2B). In particular, cellular growth, proliferation, and development were found to be most overrepresented in molecular function, which was consistent with previous studies that found the BRAF mutation activates the MAPK pathway to facilitate various cellular processes [22-24] (Figure 2B). The most significantly enriched canonical pathway was the Aryl Hydrocarbon Receptor Signaling pathway (AHR pathway, p < 3.0 × 10^−6^), which belongs to the basic helix-loop-helix/Per-Arnt-Sim family of transcription factors (Figure 2B). This pathway has been reported to regulate xenobiotic metabolizing enzymes such as cytochrome P450 and has been demonstrated to cross-talk with the MAPK pathway [25,26]. Recent studies revealed that the AHR pathway is involved in various signaling pathways that are critical to cell proliferation and differentiation, gene regulation, cell motility and migration, and inflammation [27,28]. Another particular interesting pathway that was enriched was the IL-1 signaling pathway (p = 3.7 × 10^−5^), which had been reported to be dysregulated by *BRAF*^*V600E*^ in a previous study [29]. This signaling pathway has also been shown to interact with the MAPK pathway [30,31] and contribute to multiple cancer progressions, including melanoma [32-34]. Taken together, our results indicate that *BRAF*^V600E^ may regulate many genes and pathways that are crucial for melanoma development.

### *BRAF*^V600E^ target genes mediated by MITF and TGFB1

**Figure 3:**
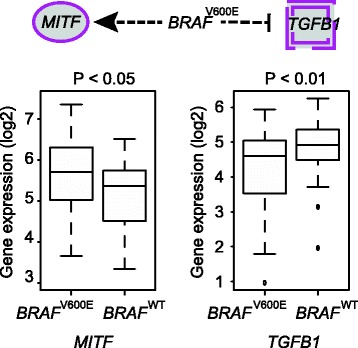
***MITF*** **and** ***TGFB1*** **dysregulated by** ***BRAF*** ^**V600E**^ **driver mutation in primary tumor samples.** Boxplots show significantly higher expressions of *MITF* but lower expressions of *TGFB1* in *BRAF* ^V600E^, as compared to *BRAF* ^WT^ samples.

We next examined whether *BRAF*^V600E^ target genes were regulated by specific up-regulators (i.e., transcription factors). The top two up-regulators identified using the IPA tool were oncogene *MITF* and tumor suppressor *TGFB1*; both were significantly enriched among *BRAF*^V600E^ target genes (p < 3.6 × 10^−16^ and p < 3.1 × 10^−9^ for *MITF* and *TGFB1*, respectively; Figure 2B). Previous studies revealed that the BRAF mutation hyper-activated the MAPK signaling pathway and led to *MITF* promotion [35-37], whereas *TGFB1* was reported to be down-regulated in multiple cancers, including melanoma [38-40]. Consistently, our results showed that *MITF* expression was also significantly higher in *BRAF*^V600E^ than in *BRAF*^WT^ samples, whereas *TGFB1* showed significantly lower expression in *BRAF*^V600E^ than in *BRAF*^WT^ (Wilcoxon test, p < 0.05 and p < 0.01 for *MITF* and *TGFB1,* respectively; Figure 3).

In addition, we repeated the Snowball analyses on TCGA melanoma metastatic samples. A total of 1010 putative *BRAF*^V600E^target genes were identified in the analysis, and we replicated a total of 213 (30%) *BRAF*^V600E^ targets from the primary tumor samples (Additional file 2). Functional enrichment analysis of up-regulators using the IPA tool revealed that TGFB1 (p = 8.89 × 10^−32^) and MITF (p = 1.51 × 10^−21^) were again significantly and consistently enriched, suggesting that gain-of-function in *BRAF*^V600E^ may generally influence the down-stream genes mediated by those two genes or pathways in different developmental stages of melanoma.

**Figure 4:**
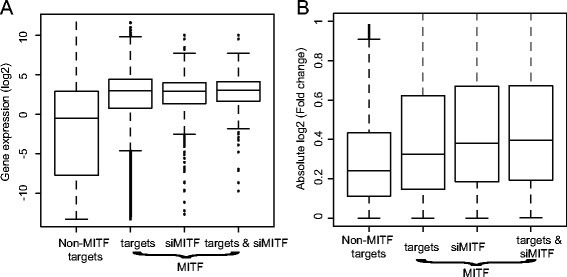
***MITF*** **and its target genes dysregulated by** ***BRAF*** ^**V600E**^ **driver mutation in primary tumor samples. A)** and **B)** Boxplots show the relative gene expression level (median value) in tumor samples and fold changes (absolute value) between *BRAF* ^V600E^ and *BRAF* ^WT^ samples for:1) non-MITF target genes, 2) MITF ChIP-Seq binding targets), 3) MITF induced genes, and 4) MITF ChIP-Seq binding and induced targets.

To further evaluate whether *BRAF*^V600E^ target genes are mostly mediated by MIT*F*, we collected 5,579 MITF target genes that were reported in a ChIP-Seq experiment and 732 *MITF*-induced targets inferred from a small interfering RNA (siRNA)-mediated *MITF* knockdown (*siMITF*) experiment in a melanoma cell line [41]. We found that genes targeted by MITF ChIP-Seq binding and si*MITF*-induced genes were more highly expressed overall than randomly selected background genes, regardless of *BRAF*^V600E^ mutation status (Figure 4A, Wilcoxon test, p < 5.0 × 10^−30^ for all comparisons). Furthermore, a random subset of non-target genes with the same range of expression levels was selected as a background to compare to MITF ChIP-Seq binding and *siMITF* induced target genes, and the result showed that both MITF ChIP-Seq binding and *siMITF* induced targets showed a significantly higher expression change in *BRAF*^V600E^ versus *BRAF*^WT^ than the randomly selected background genes (Figure 4B; Wilcoxon test, p < 3.0 × 10^−11^ for all comparisons). These results support the conclusion that *BRAF*^V600E^ leads to an increased in the level of the *MITF* gene, which likely subsequently results in the overall activation of many MITF target genes.

### Snowball identified *BRAF*^V600E^ targets in response to BRAF inhibition

**Figure 5:**
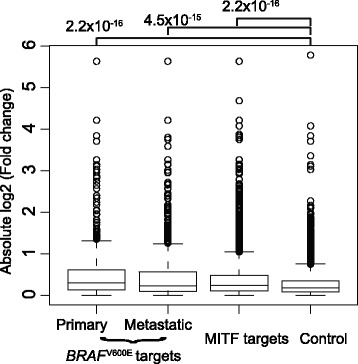
**Boxplot of gene expression fold changes (absolute value) on A375 melanoma cells before and after the treatment of vemurafenib, for Snowball identified** ***BRAF*** ^**V600E**^ **targets from both TCGA primary and metastatic tumour samples, MITF targets from literature and control genes.** P value for each comparison was derived from Wilcoxon test.

To further evaluate the identified *BRAF*^V600E^ regulated genes, we analyzed a publicly available gene expression dataset of A375 melanoma cells that harbor the *BRAF*^V600E^ mutation. This dataset contains the gene expression profiles before and after treatment with BRAF inhibitor vemurafenib (RAFi) [42]. Interestingly, we found that *BRAF*^V600E^ regulated genes identified by Snowball from both the TCGA primary and metastasis tumor samples as well as the MTIF ChIP-Seq targets [41] showed a significant response when compared to randomly selected control genes (Figure 5). This suggested that *BRAF*^V600E^ regulated genes identified by Snowball are highly reliable and *BRAF*^V600E^ may regulate MITF targets likely mediated via *MITF*.

In particular, we also found that *TGFB1* exhibited significantly elevated gene expression levels in cells with BRAF inhibitor induction, supporting that low *TGFB1* expression level is associated with *BRAF*^V600E^. However, *MITF* itself exhibited an opposite trend for gene expression. Recent work by Konieczkowski *et al.* has suggested that most drug-sensitive cell lines exhibit high *MITF* expression and activity, but this was not observed in A375 cells based on the analysis of 29 *BRAF*^V600E^-mutant melanoma cell lines [21]. This discrepancy might indicate that MITF plays a complex role in melanoma drug response.

### Global loss of DNA methylation associated with *BRAF*^V600E^

**Figure 6:**
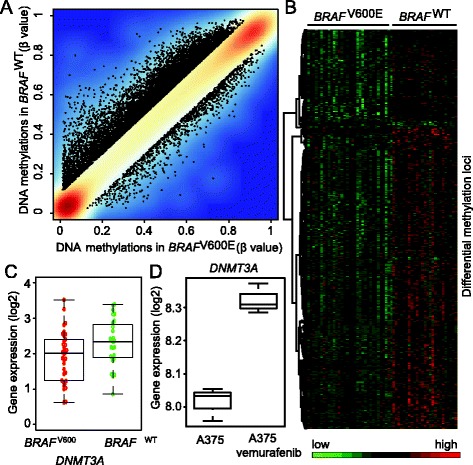
**Methylation alterations associated with** ***BRAF*** ^**V600E**^ **driver mutations in primary tumor samples. A)** Density plots of the median methylation intensity of each CpG site in *BRAF* ^V600E^ samples and *BRAF* ^WT^ samples. Methylation loci with Δβ > 0.1 were labeled with black dots. The plot indicates a global DNA methylation loss associated with BRAF^V600^. **B)** Heat-map showing differential methylation signals between *BRAF* ^V600E^ and *BRAF* ^WT^ samples, indicating a dormant methylation loss in *BRAF* ^V600E^ samples. **C)** Boxplots showing significantly lower expressions of *DNMT3A* in TCGA *BRAF* ^V600E^ versus *BRAF* ^WT^ samples. **D)** Boxplot of *DNMT3A* expression level on A375 melanoma cells before and after the treatment of vemurafenib.

We next compared DNA methylation profiles between *BRAF*^V600E^ and *BRAF*^WT^ samples (see Materials and methods). A genome-wide DNA methylation loss was observed in *BRAF*^V600E^ samples based on the comparison of DNA methylation profiles between *BRAF*^V600E^ and *BRAF*^WT^ (Figure 6A). After carrying out the differential DNA methylation analysis, we identified 523 aberrant methylation loci (i.e. CpG sites) using criteria of raw p < 1×10^−3^ and absolute intercept ≥ 0.2 (see Materials and methods). Surprisingly, 97.9% (512 of 523) showed hypomethylation in *BRAF*^V600E^ relative to *BRAF*^WT^ samples. This indicates a consistent, dominant loss of DNA methylation associated with *BRAF*^V600E^ (Figure 6B). We further repeated the same differential DNA methylation analysis on 56 *BRAF*^V600E^ versus 37 *BRAF*^WT^ TCGA metastatic samples (see Materials and methods). The same trend of genome-wide DNA hypomethylation as well as significant aberrant methylation sites was found to be associated with *BRAF*^V600E^ (Additional file 3). These results were consistent with the previous findings [43,44].

To explore the possible mechanism associated with the genome-wide hypomethylation, we systematically examined whether *BRAF*^V600E^ dysregulated any previously reported chromatin regulatory factors, such as DNA methyltransferases. Interestingly, only *DNMT3A*, functioning as a *de novo* DNA methyltransferase, showed significantly lower expression in *BRAF*^V600E^ (Figure 6C) and was identified as a putative *BRAF*^V600E^ target. In the preceding analysis, *DNMT3A* also exhibited significantly elevated expression levels in the A375 melanoma cells after being induced with the BRAF inhibitor (Figure 6D). It should be noted that no significantly differential gene expression patterns were observed based on the analysis of metastatic samples. One possible explanation is that *DNMT3A* might play a critical role in the initiation of tumorigenesis but may not be necessary in the later metastatic stage to maintain global hypomethylation. Taken together, these results suggest that *BRAF*^V600E^ might initiate genome-wide epigenetic modifications through the regulation of *DNMT3A,* facilitating the initiation of melanoma tumorigenesis [35,37].

### *BRAF*^V600E^ targets associated with melanoma proliferation

**Figure 7:**
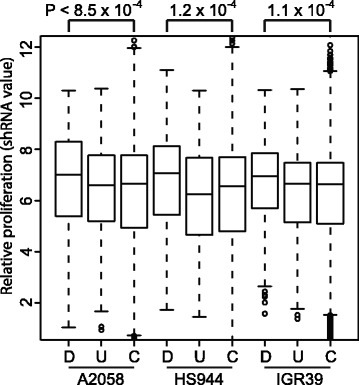
***BRAF*** ^**V600E**^ **target genes in melanoma cell proliferation.** Boxplots of shRNA values (measuring melanoma relative cell proliferation) for *BRAF* ^V600E^ target genes in down-regulation (D), up-regulation (U), and control genes (C). *BRAF* ^V600E^ target genes in down-regulations showed a statistically significant increase of melanoma cell proliferation relative to the control, whereas up-regulation genes exhibited a slight decrease in melanoma cell proliferation.

We have shown that putative *BRAF*^V600E^ target genes may play essential roles in melanoma tumorigenesis. To further verify the effects of those genes on cancer cell proliferation, we used a publicly available, large-scale gene silencing dataset from the short hairpin RNA (shRNA) screens of three melanoma cell lines (see Materials and methods, [45]). Among the 711 putative *BRAF*^V600E^ target genes, we found that down-regulated genes significantly increased cell growth and proliferation, whereas up-regulated genes slightly decreased both (Figure 7). According to the effects of putative *BRAF*^V600E^ targets on melanoma cell proliferation, we identified top tumor suppressor genes including *TGFB1, TGFB1I1, PRODH, NAT6, ZNF205, ZNF142, FRS3, RUNX3, IGFBP5, HPGD, MAPK11,* and *NFIC,* which significantly increased cell growth and proliferations. In contrast, top oncogenes including *MET, BFSP1, CDH19,* and *ST6GALNAC3* were found to be associated with decreased cell growth and proliferations*.* In summary, our results suggest that *BRAF*^V600E^ may play essential functional roles in cell growth and proliferation.

## Discussion

To our knowledge, this study is among the first attempts at an in-depth exploration of the functional consequences of a single driver mutation using an integrative genomic data analysis strategy. We applied our recently developed Snowball approach and identified 711 putative *BRAF*^V600E^ target genes, many of which are known to be involved in tumorigenesis. We further demonstrated that *BRAF*^V600E^ might dysregulate specific cancer-related pathways and epigenetic modifications in melanoma tumorigenesis.

Although previous findings based on the differential gene expression analysis of *BRAF*^V600E^ and *BRAF*^WT^ samples provided novel insights into the understanding of *BRAF*-driven biology in melanoma [14,15], applying a sensitive detection method towards the functional consequences of the driver mutation *BRAF*^V600E^ from a cohort of clinical samples is challenging. Traditional analyses used to detect significantly and differentially expressed genes are based on statistical parametric models, typically in a regression framework [46,47]. Those approaches assume expression independence among genes and apply a gene-by-gene strategy. However, analyses based on those approaches may result in ineffective detection of important gene or pathway targets due to the fact that a driver mutation is typically part of a small sample size situation and is expected to alter the expression of its cognate genes and genes in the same downstream pathways. The Snowball approach was implemented to meet the specific challenges in identifying the functional consequences of a driver mutation on clinical samples. Based on gene transcriptions and their interactions in the context of regulatory networks and the fact that a driver mutation may disturb the gene regulatory networks and generate differential co-expression profiles, the Snowball approach specifically utilizes a multivariate, distance-based regression to provide a more sensitive detection based on the co-expression profile of a given set of genes and its association with the mutation status. It is known that each tumor genome may have about 2–8 driver mutations, and their frequencies in the population are typically not high [48]. It is a real challenge to detect them using traditional differential signals. The Snowball approach is specifically developed to amplify the detection power by aggregating with resampling. Our simulation and real data analyses demonstrate that it is a more powerful approach for identifying large numbers of potential targets for downstream analyses. Additionally, the genetic background of patients and their tumor samples exhibit high heterogeneity with patient-specific and sample-specific variation. This heterogeneous predisposition to the driver mutation perturbation may lead to different gene expression patterns per gene from sample to sample as well as from patient to patient. Snowball utilizes a distance-based regression based on the gene co-expression profiles and assigns a robust ranking index to genes even when they have different predispositions [49].

This study revealed a key regulatory mechanism in melanoma, where *BRAF*^V600E^ may play dual roles as a positive regulator of the MITF pathway and as a negative regulator of the TGFB1 pathway in the initiation of melanoma development. Recently, several studies have pinpointed an alteration of MITF in patients that may fail to eradicate tumors due to chemoresistance, which reactivates the MAPK signaling pathway [50-52]. Our work, together with those findings, highlights the potentially important role of MITF in melanomagenesis. In contrast to MITF, *BRAF*^V600E^ represses the TGFB1 pathway, which may lead to the deactivation of the apoptosis process and the consequent cause of uncontrolled cell proliferation [53]. Moreover, *DNMT3A*, which also acts as a potential TGFB1 target [54], was found to be likely to mediate *BRAF*^V600E^ epigenetic modifications in this study, thus facilitating melanoma development. While these findings are insightful, future studies using *in vitro* and *in vivo* assays are warranted to verify these results.

In conclusion, we performed an integrative analysis to exhaustively interrogate mutation, expression, and methylation datasets in an attempt to detect putative target genes and their regulations that are associated with the *BRAF*^V600E^ driver mutation in melanoma. Our analyses identified not only known genes that contribute to melanoma pathogenesis but also many novel genes with potential clinical relevance. Importantly, our analysis indicated that a substantial proportion of the putative *BRAF*^V600E^ target genes were significantly regulated by the transcription factor MITF and tumor suppressor TGFB1, suggesting that *BRAF*^V600E^ may control specific cancer-related pathways via MITF and TGFB1 in order to initiate tumorigenesis. In particular, *DNMT3A*, one of the putative *BRAF*^V600E^ targets, may reprogram epigenetic modifications to facilitate cancer development. These target genes were further shown to be essential in melanoma cell proliferation. Our analysis strategy provides a novel way to explore the functional consequences of a driver mutation and can be similarly applied to other driver mutations in complex diseases.

## Materials and methods

### Datasets

We retrieved genomic data, including somatic mutations, from whole exome sequencing (total 385 samples), DNA methylation (n = 413), and RNA-Seq (n = 371) of cutaneous melanoma samples from the TCGA data portal (https://tcga-data.nci.nih.gov/tcga/). We analyzed only those samples derived from primary tumors with matched mutation, expression, and methylation profiles in each sample. The DNA somatic mutation data (TCGA level 2) was retrieved from the TCGA somatic mutation annotation file (summarized as “maf” file), from which the *BRAF*^V600E^ mutation status of each sample was examined and determined. We systemically examined the mutational profiles across all TCGA melanoma samples (N = 385). Since multiple driver mutations may co-exist on the same sample, the samples with known driver mutations including *NRAS*, *CDKN2A*, *GNAQ*, *KIT* and *GNA11* have been removed from both the case and negative control groups to reduce the confounding effects. We finally included a dataset of 34 *BRAF*^V600E^ samples and 27 *BRAF*^WT^ samples; here, wide-type (WT) denotes pan-negative samples (those without any mutations in the above driver genes) (Figure 1). The gene-level expression data (TCGA level 3) was generated using Illumina HiSeq 2000 and measured by normalized RSEM (RNA-Seq by Expectation-Maximization) read counts. The DNA methylation data (TCGA level 3) was generated using the Illumina HumanMethylation450 BeadChip Array. Each methylation CpG locus was measured by a β value representing a ratio of M/(U + M), where M is the methylated probe intensity and U is the unmethylated probe intensity. The β value ranged from 0 to 1 (0: unmethylated; 1: fully methylated).

The transcription factor MITF’s binding targets from the ChIP-Seq data and its induced genes detected by the small interfering RNA (siRNA)-mediated *MITF* knockdown (*siMITF*) experiment were collected from a previous work [41].

### DNA methylation analysis

We started methylation analysis from methylation profiles (β value, TCGA level 3) and then converted methylation β value to M value, which is compatible with the typical assumptions of linear models. We applied the R package Minfi [55] to detect differential methylation loci between *BRAF*^V600E^ and *BRAF*^WT^ samples. Significantly differentially methylated sites were detected used an F-test implemented in the function ‘dmpFinder’. The significantly aberrant methylation loci were identified by applying raw p value < 1 × 10^−3^ and absolute intercept ≥ 0.2.

### Gene expression analysis

We developed the Snowball algorithm to identify a set of genes or gene modules that are likely regulated by a driver mutation [21]. This approach takes into account gene-gene interactions by evaluating each gene in a group of other genes. It is a more effective learning approach for the identification of functionally relevant genes or gene modules medicated by driver mutations that spread their genetic turbulence in the gene regulatory network to penetrate its functional impact. By applying the Snowball approach to the *BRAF*^V600E^ and *BRAF*^WT^ sample sets, we identified 1072 genes with significantly aggregated association with the mutation *BRAF*^V600E^. We further applied the Weighted Gene Co-expression Network Analysis [48] and identified 9 gene modules, each significantly associated with the BRAF mutation status when assessed using a generalized distance-based regression [21] at a permutation P < 0.05. The fold change of each gene’s expression in *BRAF*^V600E^ relative to *BRAF*^WT^ was calculated based on log2-transformed RSEM measurement.

To evaluate how Snowball identified BRAF regulated genes in response to the BRAF inhibitor vemurafenib, we also analyzed gene expression data on A375 melanoma cells harboring the *BRAF*^V600E^ mutation from recent literature. This dataset contained the gene expression profiles before and after treatment with the BRAF inhibitor (GEO: GDS5085) [6]. Briefly, using LIMMA, we compared the gene expression profiles of A375 melanoma cells before and after treatment with the BRAF inhibitor, and the fold change of each gene was computed and reported. A total of 5000 genes were randomly selected from the genome as control genes for the comparative analysis.

### Functional analysis

For the abovementioned 711 putative *BRAF*^V600E^ regulated target genes, we examined their functional enrichment in gene networks and biological pathways, using the Ingenuity Pathway Analysis (IPA) tool (http://www.ingenuity.com/). The top 5 ranked gene networks and biological pathways were present.

### Effect of gene silencing on cell proliferation using RNA interference data

To estimate the effect of an individual gene on cancer cell proliferation, we downloaded a comprehensive dataset from a genome-wide shRNA analysis of 10,941 genes (comprising of 52,209 probes) for three melanoma cell lines: A2058, HS944, and IGR39 (from the previous study) [45]. The effect of an individual gene’s silence for each of the three melanoma cell proliferations (measured by shRNA value) was computed using the log2 ratio of cell abundance in the pool generated by shRNA sequences at the endpoint, relative to the initial reference pool (details described in [45]).

## Additional files

Additional file 1:
**A total of 711** ***BRAF*** ^**V600E**^ **target genes were listed based on the analysis of primary samples.**
Additional file 2:
**A total of 1010** ***BRAF*** ^**V600E**^ **target genes were listed based on the analysis of primary samples.**
Additional file 3:
**Methylation alterations associated with** ***BRAF*** ^**V600E**^ **driver mutations based on TCGA metastatic samples.** A) Density plots of the median methylation intensity of each CpG site in *BRAF* ^V600E^ samples and *BRAF* ^WT^ samples. Methylation loci with Δβ > 0.1 were labeled with black dots. B) Heat-map showing differential methylation signals between *BRAF* ^V600E^ and *BRAF* ^WT^ samples, indicating a dominant methylation loss in *BRAF* ^V600E^ samples.
