# Supplementary figures and images for: In-depth genomic data analyses revealed complex transcriptional and epigenetic dysregulations of BRAFV600E in melanoma
Source: Mol Cancer. 2015 Mar 14;14:60. doi: 10.1186/s12943-015-0328-y (PMC4373107; doi:10.1186/s12943-015-0328-y)

A

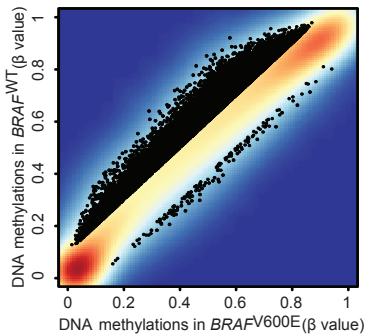

B

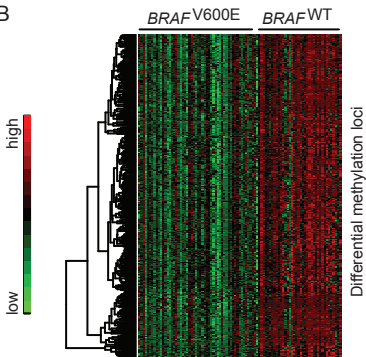

Supplement: Additional file 3: — Methylation alterations associated with BRAF V600E driver mutations based on TCGA metastatic samples. A) Density plots of the median methylation intensity of each CpG site in BRAF V600E samples and BRAF WT samples. Methylation loci with Δβ > 0.1 were labeled with black dots. B) Heat-map showing differential methylation signals between BRAF V600E and BRAF WT samples, indicating a dominant methylation loss in BRAF V600E samples. [file 12943_2015_328_MOESM3_ESM.pdf]
